# Impacts of weaning weights and mycotoxin challenges on jejunal mucosa-associated microbiota, intestinal and systemic health, and growth performance of nursery pigs

**DOI:** 10.1186/s40104-022-00691-6

**Published:** 2022-04-13

**Authors:** D. M. Holanda, S. W. Kim

**Affiliations:** Department of Animal Science, North Carolina State University, Raleigh, 27695 USA

**Keywords:** Growth, Health, Mycotoxins, Nursery pig, Weaning weight

## Abstract

**Background:**

This study aimed at investigating the effects of mycotoxin challenge on the growth and physiology of nursery pigs with different weaning weights.

**Results:**

At weaning, 10 pigs were euthanized to collect jejunal mucosa and 90 pigs were assigned following a randomized complete block design in a 2 × 2 factorial arrangement of treatments with 3 pigs per pen. Factors were: weaning weight (light: body weight, BW < 7.5 kg or heavy: BW > 9.0 kg); and dietary mycotoxins (supplementation of 0.2 mg/kg aflatoxins, 2.0 mg/kg deoxynivalenol). All diets had titanium dioxide as an external marker at 0.5%. Growth performance and fecal score were recorded until pigs achieved 20 kg BW (light pigs average BW = 21.1 kg and heavy pigs average BW = 20.5 kg). Pigs were sampled for blood, ileal digesta, jejunal tissue and mucosa at 20 kg BW. Data were analyzed using the mixed procedure of SAS. At weaning, light pigs had decreased (*P* < 0.05) jejunal interleukin-8, increased (*P* < 0.05) tumor necrosis factor-α, and increased (*P* < 0.05) α-diversity indexes of jejunal mucosa-associated microbiota. At 20 kg of BW, light pigs had decreased (*P* < 0.05) average daily gain (ADG), average daily feed intake (ADFI), and gain to feed ratio (G/F). Mycotoxins decreased (*P* < 0.05) BW, ADG, ADFI, and G/F. Light pigs tended to have increased fecal score on d 0 (*P* = 0.080), d 10 (*P* = 0.069), and increased (*P* < 0.05) fecal score at 20 kg. Mycotoxins decreased the apparent ileal digestibility of nitrogen (*P* < 0.05). Light pigs had increased (*P* < 0.05) intestinal malondialdehydes and interleukin 8. Mycotoxins tended to increase (*P* = 0.060) intestinal tumor necrosis factor-α.

**Conclusions:**

Nursery pigs with light weaning weight were more susceptible to jejunal inflammation and had impaired intestinal health due to weaning stress, whereas mycotoxins diminished the health and growth of nursery pigs regardless of weaning weight.

## Introduction

Weaning events such as changes in diets, facilities, and littermates are among the factors that cause weaning stress. The weaning stress can lead to inflammatory activation in the intestine and damage of enterocytes [1, 2], reducing the digestibility of nutrients in feeds and nutrient absorption. It has long been demonstrated that pigs weaned at older ages are less susceptible to weaning stress and, thus, have improved feed intake and body weight gain [3–5]. More recently, weaning stress was found linked to long-lasting or permanent detrimental effects on the gastrointestinal tract and the immune system of pigs [4]. Also, weaning weight, as an estimate of weaning age [6], can affect the pig intestinal microbiota [7] as well as health and growth during the nursery period [4].

Mycotoxins are prevalent in most feedstuffs fed to pigs. Aflatoxins and deoxynivalenol are mycotoxins produced by filamentous fungi that may potentially contaminate feeds and cause detrimental effects in pigs. Aflatoxins and deoxynivalenol impair protein synthesis [8, 9], cause inflammatory and oxidative stress responses [10–12], alter intestinal microbiota [13, 14], and reduce energy and nutrient digestibility [15]. Thus, pigs challenged with mycotoxins have impaired intestinal health, reduced nutrient utilization [15, 16], and hepatic damage [17, 18], resulting in reduced growth performance [14–16].

It has been shown that the susceptibility of pigs to the toxic effects of mycotoxins decreases with age [14]. It is hypothesized that pigs with low weaning weight may have greater susceptibility to mycotoxins with impaired intestinal health and modification of microbiota. This study investigated the effects of supplemental 0.2 mg/kg of aflatoxins and 2.0 mg/kg of deoxynivalenol in feeds on growth performance, health status, and intestinal microbiota of nursery pigs with different body weights at weaning.

## Materials and methods

The experiment was carried out at the North Carolina State University Swine Evaluation Station (Clayton, NC, USA). A protocol for this experiment was approved by the Institutional Animal Care and Use Committee at North Carolina State University.

### Animals and diets

One hundred and six pigs were selected from 186 newly-weaned pigs (28 days of age) to form a 2-category factor. This factor was based on weaning weight: (i) L, light pigs with weaning weight equal or reduced to 7.5 kg (6.93 ± 0.07 kg); (ii) H, heavy pigs with weaning weight equal or above 9.0 kg (9.79 ± 0.14 kg). Such weaning weights were selected based on the body weights of piglets weaned at 21 (7.5 kg) or 28 (9.0 kg) days of age. However, the birthdates, and consequently the weaning age of the pigs in the current study, were not recorded. Therefore, this study will refer only to weaning weight as a reference to weaning age. Within each weaning weight category, pigs were ranked by body weight into three body weight groups (high, intermediate, and low weaning weight). Subjects with the highest (*n* = 2, 1 male and 1 female), intermediate (*n* = 2, 1 male and 1 female), and lowest (*n* = 1, female) weaning weights within L and H categories were selected (totaling *n* = 10). The absence of male subjects selected for the lowest weaning weight groups within each category happened due to a lack of males with weaning weights suitable for such groups. Therefore, it was arranged to keep the same number of males and females in each treatment for the remaining of the experimental period. The selected pigs were euthanized by penetration of captive bolt followed by exsanguination for sample collection. The remaining 96 selected pigs were assigned following a randomized complete block design (using sex as a block) to 4 treatments based on a 2 × 2 factorial arrangement with three pigs per pen, totaling 32 pens, 16 replicates per factor, and 8 replicates per treatment. First factor was body weight at weaning as described above: either less than 7.5 kg greater than 9.0 kg. The second factor was supplemental mycotoxins: a control diet without supplemental mycotoxins or a diet with an additional 2 mg/kg of deoxynivalenol and 0.18 mg/kg of aflatoxins. Additional mycotoxin contamination was achieved by the inclusion of deoxynivalenol-contaminated corn DDGS and aflatoxin-contaminated corn. Pigs were fed phase 1 diets from the beginning of the trial (d 0) until achieving 11 kg of body weight and phase 2 diets from 11 to 20 kg of body weight. Because of different weaning weights, phase 1 lasted 14 d for the L group and 4 d for the H group; while phase 2 lasted 17 d for both weaning weight groups. Diets were formulated to meet or exceed the NRC [19] requirements for nursery pigs (Table 1). At the end of phase 2, 24 pigs were selected based on sex (half male and half female) and by treatment, one pig per pen with the median body weight was selected from the heavier, intermediate, and lighter pens. The 24 selected pigs were euthanized for sampling following the same procedure as described previously for d 0.

**Table 1:** Composition of experimental diets

Dietary phase	1		2	
Mycotoxin	–	+	–	+
Feedstuff, %				
Corn, yellow dent	29.11	23.11	40.87	34.84
AF corn^1^	0.00	6.00	0.00	6.00
Corn DDGS	22.00	0.00	22.00	0.00
DON corn DDGS	0.00	22.00	0.00	22.00
Soybean meal	15.56	15.56	26.50	26.50
Whey permeate	20.00	20.00	5.00	5.00
Poultry meal	4.00	4.00	0.00	0.00
Blood plasma	5.00	5.00	0.00	0.00
Limestone	1.19	1.19	1.10	1.10
Dicalcium phosphate	0.08	0.08	0.60	0.60
L-Lys HCl	0.48	0.48	0.42	0.42
DL-Met	0.11	0.11	0.07	0.07
L-Thr	0.07	0.07	0.07	0.07
Salt	0.22	0.22	0.22	0.22
Mineral supplement	0.15	0.15	0.15	0.15
Vitamin supplement	0.03	0.03	0.03	0.03
Poultry fat	2.00	2.00	2.50	2.50
Titanium dioxide^2^	0.00	0.00	0.50	0.50
Calculated composition				
ME, kcal/kg	3423	3423	3397	3397
SID Lys, %	1.35	1.35	1.23	1.23
Ca, %	0.80	0.80	0.70	0.70
Aflatoxins, mg/kg	0.00	0.18	0.00	0.18
Deoxynivalenol, mg/kg	0.00	2.00	0.00	2.00

^1^ AF corn, corn contaminated with aflatoxins at 3 mg/kg. Corn DDGS, corn distillers dried grains with solubles. DON corn DDGS, either normal or contaminated with deoxynivalenol. *ME*, metabolizable energy. *SID*, standardized ileal digestibility

^2^ Titanium dioxide was added at 0.5% to phase 2 diets as an external marker for assessment of apparent ileal digestibility of nutrients in feed

### Sample collection and processing

Feed samples were randomly collected from each dietary treatment and phase totaling 2 kg each. Subsamples of 300 g were sent for proximate analysis at the North Carolina Department of Agriculture (Raleigh, NC, USA). Subsamples of 2 g were ground and sent to the Veterinary Diagnostic Laboratory (North Dakota State University, Fargo, ND, USA) for determining concentrations of mycotoxins by liquid chromatography-tandem mass spectrometry.

The fecal score was assessed by observing feces on the floor according to a scale ranging from 1 to 5 [20] in the morning (0700 h) of d 0, 3, 5, 7, 10, 15, and 20. Pen floors were cleaned in the afternoon before each assessment.

The 10 pigs euthanized at the beginning of the study and the 24 pigs euthanized at the end of the study were sampled for jejunal tissues and scrapped mucosa from the duodenum and proximal jejunum [21]. The 10 pigs sampled at the beginning of the study were feed-fasted for approximately 4 h, during transportation to research farm (feed and water deprived) and animal allotment (feed deprived), before collections. The pigs sampled at the end of the study had free access to water but were 6-h fasted on the day before sampling and re-fed 8 h preceding sampling time. One 5 cm-long section of each duodenal and jejunal tissue was fixed in buffered formalin at room temperature for 72 h. After that, 2 cross-sections of 0.5 cm of each sample were moved to cassettes and transferred to 70% ethanol. The cross-sections were sent to the North Carolina State University Histopathology Laboratory (College of Veterinary Medicine, Raleigh, NC, USA) for Ki-67 staining according to procedures described by Kim et al. [14]. Intestinal mucosa was sampled by scraping intestinal sections of 15 cm. The scrapped mucosa was placed in vials and immediately stored in liquid nitrogen for further assessment of immune and oxidative stress markers. One more vial of proximal jejunal mucosa was obtained for microbiome sequencing and placed in liquid nitrogen. The vials were transferred, by the end of the day, to a − 80 °C freezer. Duodenum and proximal jejunum were chosen as the sampling sites because deoxynivalenol is readily absorbed by paracellular diffusion in the proximal small intestine. Thus, these sites seem to be more affected in pigs fed diets with deoxynivalenol [16].

The 24 pigs euthanized at the end of the study were also sampled for blood and ileal digesta. Blood (10 mL) was collected in tubes (Becton Dickinson Vacutainer Systems, Franklin Lakes, NJ, USA) by puncturing the jugular vein (0.8mm × 32 mm needles, Eclipse, Becton Dickinson Vacutainer Systems) for performing serum analyses of biochemical variables and electrolytes. Blood samples were allowed to clot at 4 °C for 4 h before centrifuging at 1500×*g* at 4 °C for 15 min (5811F, Eppendorf, Hamburg, HH, Germany). The supernatant, blood serum, was transferred to duplicates of 1.5-mL vials (Fisherbrand, Fisher Scientific, Hampton, NH, USA) and stored at − 80 °C freezer (812660–760, Thermo Fisher Scientific, Waltham, MA, USA) until laboratory analyses. The ileal digesta was collected by gradually squeezing the ileal content delimited proximally by the ileocecal fold and caudally by the ileocecal junction. Ileal digesta containers were immediately immersed in ice and then stored at − 20 °C until further analyses to estimate the ileal digestibility of dry matter, protein, gross energy, and ether extract contents in the feed.

### Assay procedures

Histology sections stained for antibody of Ki-67 protein were measured for villus height (from the tip of the villus to the villus-crypt junction), villus width (perpendicular line to the longer axis of the villus at one-half of the villus height), and crypt depth (from villus junction to the base of the crypt) were taken from well-oriented intact villus and its associated crypt [11]. The average among 10 measurements was used for each pig. The estimated proliferative rate was obtained by calculating the proportion of Ki-67-positive cells to the total cell number in the crypt by using ImageJS software [22]. A single evaluator executed all the histological analyses for intestinal morphometry and Ki-67 counting.

One vial of duodenal and proximal jejunal mucosa was used for assessing immune and oxidative stress markers in each intestinal section. The markers were evaluated by quantifying protein carbonyls (STA-310, Cell Biolabs, Inc., San Diego, CA, USA), malondialdehydes (STA-330, Cell Biolabs, Inc.), tumor necrosis factor-alpha (PTA00, R&D Systems, Inc., Minneapolis, MN, USA), interleukin-8 (P8000, R&D Systems, Inc.), immunoglobulin A (E100–102, Bethyl Laboratories, Inc., Montgomery, TX, USA), and immunoglobulin G (E100–104, Bethyl Laboratories, Inc.) relative to the protein content in samples (PierceTM BCA Protein Assay Kit, Thermo Fisher Scientific). First, cellular content was extracted by thawing vials on ice and homogenizing (Tissuemiser, Thermo Fisher Scientific) 1 g of the sample with 2 mL of phosphate-buffered saline (MP Biomedicals, Inc., Santa Ana, CA, USA) for 30 s. The supernatant obtained after centrifuging at 87,000×*g* for 20 min were placed in individual vials to be used for measuring each of the markers and stored at − 80 °C until further use. The manufacturer’s manual for each kit was followed in the laboratory assays as described by Holanda et al. [15].

The second vial of proximal jejunal mucosa was used to assess the diversity and relative abundance of mucosa-associated microbiota. The DNA was extracted from proximal jejunal mucosa with QIAGEN’s QIAamp® DNA Stool MiniKit (Qiagen, Crawley, SXW, UK). The DNA samples were sent to Mako Medical Laboratories (Raleigh, NC, USA) for microbial sequencing using the 16S rRNA technique. The Ion Chef instrument was used to prepare the samples for the template and sequencing was performed on the Ion S5 system (Thermo Fisher Scientific, Inc.). Variable regions V1, V2, V3, V4, V6, V7, V8, and V9 of the 16S rRNA gene were amplified with the Ion 16S Metagenomics Kit (Thermo Fisher Scientific, Inc.). The hypervariable regions were processed using the Torrent Suite Software (version 5.2.2; Thermo Fisher Scientific, Inc.) to produce raw unaligned sequence data files for further analysis. Sequence data analysis, alignment to GreenGenes and MicroSeq databases, alpha diversity plot generation, and operational taxonomic unit (OTU) table generation were performed by the Ion Reporter Software Suite of bioinformatics analysis tools (version 5.2.2; Thermo Fisher Scientific, Inc.). Samples were analyzed using Ion Reporter’s Metagenomics 16S workflow powered by Qiime (Quantitative Insights Into Microbial Ecology, version w1.1). Alpha-diversity was estimated with 3 indexes: Chao1, Shannon-Weaver, and Simpson.

Serum samples were submitted for biochemical profiling at ANTECH Diagnostic Laboratory (Cary, NC, USA). Ileal digestibility of dry matter [23], protein (828 Series Carbon/Nitrogen Analyzer with Cornerstone Brand Software, FP828, Model Number 622–100-700; LECO, St. Joseph, MI, USA), gross energy (6200 Calorimeter, Parr Instrument Company, Moline, IL, USA), and ether extract (method 920.39, [24]) were evaluated relative to their contents in feed using titanium dioxide as an external marker.

### Statistical methods

The use of phases based on body weight of pigs aimed to enable the comparison for the body weight factor at different time points during the study. Also, the phase feeding program was followed to simulate a commercial scenario and to enable comparisons to previous publications [25–28].

For the statistical analysis, SAS 9.3 software (Cary, NC, USA) was used to process data with factors and their interaction as fixed effects and sex block as a random effect using the MIXED procedure. In case of interaction, treatments were compared with the PDIFF statement and tested by Tukey test. The pen was considered the experimental unit for growth performance and fecal score data. The pig, representing each pen, was considered the experimental unit for all other variables. Results were considered statistically different for *P* < 0.05 and considered a tendency for 0.05 ≤ *P* < 0.10.

## Results

In the finished diets, the mycotoxin concentrations in the negative control diet (−) and the diet with additional mycotoxins (+) for aflatoxins were 0.02 and 0.21 mg/kg and for deoxynivalenol were 0.520 and 2.32 mg/kg, respectively (Table 2). The resultant mycotoxin concentrations for additional aflatoxins and deoxynivalenol are close to the concentrations expected, an additional 0.19 mg/kg of aflatoxins and an additional 1.80 mg/kg of deoxynivalenol. Of note, two pigs were removed from the study, one on d 11 due to neurological signs, and the other on d 18 because of severe diarrhea. Both were light pigs, the first was receiving the diet with additional mycotoxin contamination and the second was not.

**Table 2:** Mycotoxin concentrations in diets fed to pigs based on a 2-phase feeding program

Dietary phase^1^	1		2	
Mycotoxin	–	+	–	+
Aflatoxin B1, mg/kg	< 0.020	0.130	< 0.020	0.242
Aflatoxin B2, mg/kg	< 0.020	< 0.020	< 0.020	0.033
Deoxynivalenol, mg/kg	0.472	2.258	0.566	2.389
Fumonisin B1, mg/kg	0.326	1.810	0.598	2.000
Fumonisin B2, mg/kg	< 0.200	0.378	< 0.200	0.445
Zearalenone, mg/kg	< 0.100	0.159	< 0.100	0.171

^1^Dietary treatments were based on 2 phases: phase 1 from d 0 until animals achieved 11 kg of body weight (4 d for pig weaning weight > 9 kg and 14 d for pig weaning weight <  7.5 kg) and phase 2 until pigs achieved 20 kg of body weight (17 d for both weaning weight groups). Concentrations determined by mycotoxin screen by liquid chromatography-tandem mass spectrometry (Veterinary Diagnostic Laboratory, North Dakota State University, Fargo, USA)

Light pigs had reduced (*P* < 0.05) body weight on d 0, as expected, and by the end of phase 1 (Table 3). Pigs fed mycotoxins had reduced (*P* < 0.05) body weight by the end of phase 2. Among light pigs feeding mycotoxins tended to have reduced (*P* = 0.070) body weight on d 31. Light pigs had reduced (*P* <0.05) average daily gain (ADG) during phase 1 but increased ADG during phase 2 than heavy pigs. Light pigs tended to have reduced (*P* = 0.054) ADG in the overall period than heavy pigs. Pigs fed mycotoxins had reduced (*P* < 0.05) ADG during phase 2 and in the overall period. Light pigs had reduced (*P* < 0.05) average daily feed intake (ADFI) during phase 1 and in the overall period, but increased (*P* < 0.05) ADFI during phase 2 than heavy pigs. Pigs fed mycotoxins had reduced (*P* < 0.05) ADFI during phase 2 and in the overall period. Pigs fed mycotoxins tended to have an increased (*P* = 0.055) gain to feed ratio (G/F) in the overall period.

**Table 3:** Growth performance of pigs with different weaning weights and mycotoxin challenges

Weaning weight (WW)	< 7.5 kg		> 9.0 kg		SEM	*P-*value		
Mycotoxin^1^ (MTX)	–	+	–	+		WW	MTX	WW vs. MTX
Body weight, kg								
d 0	6.9	6.9	9.8	9.8	0.2	< 0.001	0.974	0.961
Phase 1	10.5	10.1	11.3	11.1	0.2	0.029	0.435	0.756
Phase 2	22.4	19.8	21.2	19.7	0.8	0.422	0.015	0.508
ADG, g								
Phase 1	257	228	371	326	32	0.002	0.259	0.800
Phase 2	686	572	584	508	34	0.006	0.002	0.524
Overall	498	416	544	473	30	0.054	0.006	0.836
ADFI, g								
Phase 1	373	316	532	498	32	< 0.001	0.168	0.727
Phase 2	1038	807	842	719	50	< 0.001	< 0.001	0.129
Overall	737	585	783	677	38	0.033	< 0.001	0.452
G/F								
Phase 1	0.70	0.70	0.70	0.66	0.05	0.639	0.615	0.648
Phase 2	0.67	0.71	0.69	0.71	0.02	0.687	0.119	0.444
Overall	0.67	0.71	0.68	0.70	0.01	0.945	0.055	0.469

^1^Dietary treatments were based on 2 phases: phase 1 from d 0 until animals achieved 11 kg of body weight (4 d for pig weaning weight > 9 kg and 14 d for pig weaning weight <  7.5 kg) and phase 2 until pigs achieved 20 kg of body weight (17 d for both weaning weight groups).*ADG*, average daily gain; *ADFI*, average daily feed intake; *G/F*, gain to feed ratio

For fecal scores, light pigs tended to present increased fecal score on d 0 (*P* = 0.080) and d 10 (*P* = 0.069; Table 4). On d 15, there was a tendency for interaction (*P* = 0.092) where light pigs fed mycotoxins tended to have increased (*P* = 0.096) fecal score than heavy pigs fed diets without additional mycotoxins. Light pigs presented an increased (*P* < 0.05) fecal score on d 20. There was no difference (*P* > 0.05) in the fecal score on d 3, 5, or 7 among pigs.

**Table 4:** Fecal score observed in pigs with different weaning weights and mycotoxin challenges

Weaning weight (WW)	< 7.5 kg		> 9.0 kg		SEM	*P-*value		
Mycotoxin (MTX)	–	+	–	+		WW	MTX	WW vs. MTX
d 0	2.25	2.25	1.63	1.13	0.48	0.080	0.607	0.607
d 3	2.25	2.25	1.88	1.75	0.41	0.294	0.880	0.880
d 5	2.50	3.13	2.75	2.75	0.43	0.886	0.476	0.476
d 7	2.13	2.38	2.25	2.38	0.54	0.909	0.733	0.909
d 10	2.25	2.50	1.25	2.00	0.40	0.069	0.217	0.532
d 15	2.63^a^	1.88^aX^	1.00^bY^	1.50^b^	0.36	0.010	0.730	0.092
d 20	1.88	1.63	1.38	1.00	0.29	0.020	0.179	0.785

Dietary treatments were based on 2 phases: phase 1 from d 0 until animals achieved 11 kg of body weight (4 d for pig weaning weight > 9 kg and 14 d for pig weaning weight < 7.5 kg) and phase 2 until pigs achieved 20 kg of body weight (17 d for both weaning weight groups). ^a,b^ Means within a row with a different superscript differ (*P* < 0.05). ^X,Y^ Means within a row with a different superscript tend to differ (0.05 < *P* < 0.10)

Pigs fed mycotoxins tended to have reduced (*P* = 0.078) dry matter apparent ileal digestibility (Table 5). Pigs fed mycotoxins had reduced (*P* < 0.05) nitrogen apparent ileal digestibility. There was a tendency for interaction (*P* = 0.053) where feeding mycotoxins among light pigs increased (*P* < 0.05) ether extract apparent ileal digestibility, whereas there was no mycotoxin effect among heavy pigs. There was no difference (*P* > 0.05) in gross energy apparent ileal digestibility among pigs.

**Table 5:** Apparent ileal digestibility of nutrients in pigs with different weaning weights and mycotoxin challenges at 20 kg

Weaning weight (WW)	< 7.5 kg		> 9.0 kg		SEM	*P-*value		
Mycotoxin^1^ (MTX)	–	+	–	+		WW	MTX	WW × MTX
Dry matter, %	65.9	62.1	67.2	58.8	3.3	0.768	0.078	0.483
Gross energy, %	66.1	63.5	67.3	59.3	3.3	0.661	0.126	0.425
Nitrogen, %	79.2	69.7	79.0	68.5	2.8	0.813	0.002	0.858
Ether extract, %	73.0^b^	87.3^a^	77.9^ab^	74.5^b^	4.3	0.370	0.215	0.053

^1^Dietary treatments were based on 2 phases: phase 1 from d 0 until animals achieved 11 kg of body weight (4 d for pig weaning weight > 9 kg and 14 d for pig weaning weight < 7.5 kg) and phase 2 until pigs achieved 20 kg of body weight (17 d for both weaning weight groups). ^a,b^ Means within a row with a different superscript differ (*P* < 0.05)

Light pigs had increased (*P* < 0.05) blood serum alkaline phosphatase and reduced (*P* < 0.05) chloride (Table 6). Pigs fed mycotoxins had reduced (*P* < 0.05) albumin and albumin to globulin ratio. There was a tendency for interaction (*P* = 0.091) where among light pigs feeding mycotoxins increased globulin, whereas there was no difference among heavy pigs. There was an interaction (*P* < 0.05) where there was no difference among light pigs, whereas among heavy pigs feeding mycotoxins tended to increase (*P* = 0.093) creatinine. There was a tendency for interaction (*P* = 0.053) where among heavy pigs feeding mycotoxins increased (*P* < 0.05) BUN to creatinine ratio, whereas there was no difference among light pigs. There was an interaction (*P* < 0.05) where among heavy pigs feeding mycotoxins decreased (*P* < 0.05) phosphorus, whereas there was no difference among light pigs. There was no difference (*P* > 0.05) observed for other blood variables among pigs.

**Table 6:** Serum variables observed in pigs at 20 kg with different weaning weights and mycotoxin challenges

Weaning weight (WW)	< 7.5 kg		> 9.0 kg		SEM	*P-*value		
Mycotoxin^1^ (MTX)	–	+	–	+		WW	MTX	WW × MTX
Liver health								
Total protein, g/dL	5.05	5.08	5.07	4.88	0.15	0.496	0.576	0.422
Albumin, g/dL	3.28	2.95	3.07	2.93	0.14	0.311	0.051	0.383
Globulin, g/dL	1.77^b^	2.13^a^	2.00^ab^	1.95^ab^	0.12	0.833	0.192	0.091
Albumin/Globulin	1.88	1.43	1.57	1.52	0.12	0.340	0.050	0.110
AST, IU/L	34.8	32.2	35.2	41.2	5.3	0.386	0.755	0.420
ALT, IU/L	19.3	17.5	19.7	18.7	1.4	0.587	0.310	0.762
AST/ALT	1.80	1.90	1.81	2.16	0.25	0.587	0.370	0.616
ALP, IU/L	277	263	204	241	23	0.049	0.635	0.275
CPK, IU/L	884	884	849	910	244	0.985	0.901	0.903
Cholesterol, mg/dL	81.5	82.0	76.2	74.0	5.6	0.194	0.868	0.791
BUN, mg/dL	12.0	11.7	12.7	10.7	0.9	0.861	0.230	0.386
Creatinine, mg/dL	0.63^b^	0.58^b^	0.65^abY^	0.72^aX^	0.03	0.011	0.758	0.041
BUN/Creatinine	19.0^a^	20.2^a^	19.3^a^	14.8^b^	1.4	0.085	0.240	0.053
Glucose, mg/dL	113	111	109	102	5	0.166	0.410	0.653
Electrolytes								
Phosphorus, mg/dL	9.17^ab^	9.78^a^	9.78^a^	8.67^b^	0.34	0.470	0.470	0.019
Calcium, mg/dL	10.8	10.8	10.7	10.5	0.2	0.339	0.705	0.644
Sodium, mEq/L	145	145	145	145	1	0.445	0.445	0.645
Potassium, mEq/L	5.52	5.45	5.77	5.48	0.28	0.466	0.369	0.576
Na/K	26.3	26.8	25.2	26.8	1.3	0.528	0.247	0.528
Chloride, mEq/L	102	102	105	105	1	0.007	0.911	0.911

^1^Dietary treatments were based on 2 phases: phase 1 from d 0 until animals achieved 11 kg of body weight (4 d for pig weaning weight > 9 kg and 14 d for pig weaning weight < 7.5 kg) and phase 2 until pigs achieved 20 kg of body weight (17 d for both weaning weight groups). *AST*, aspartate aminotransferase; *ALT*, alanine aminotransferase; *ALP*, alkaline phosphatase; *BUN*, blood urea nitrogen; *CP*K, creatine phosphokinase. ^a, b^ Means within a row with a different superscript differ (*P* < 0.05). ^X, Y^ Means within a row with a different superscript tend to differ (0.05 ≤ *P* < 0.10)

**Fig. 1:**
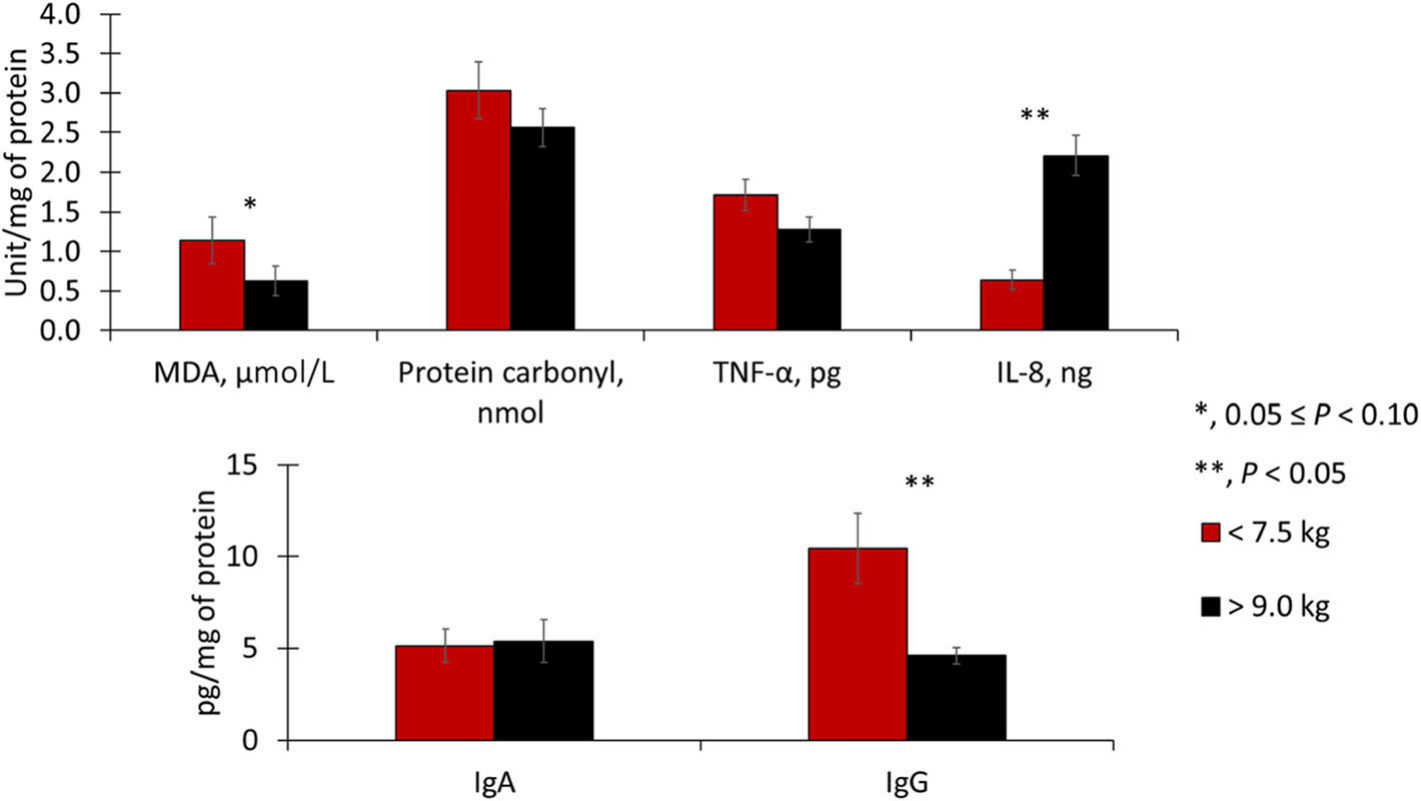
Duodenal oxidative stress, immune markers in pigs with different body weights at weaning. MDA, malondialdehydes; TNF-α, tumor necrosis factor-alpha; IL-8, interleukin 8; IgA, immunoglobulin A; IgG, immunoglobulin G, VH, villus height; CD, crypt depth

**Fig. 2:**
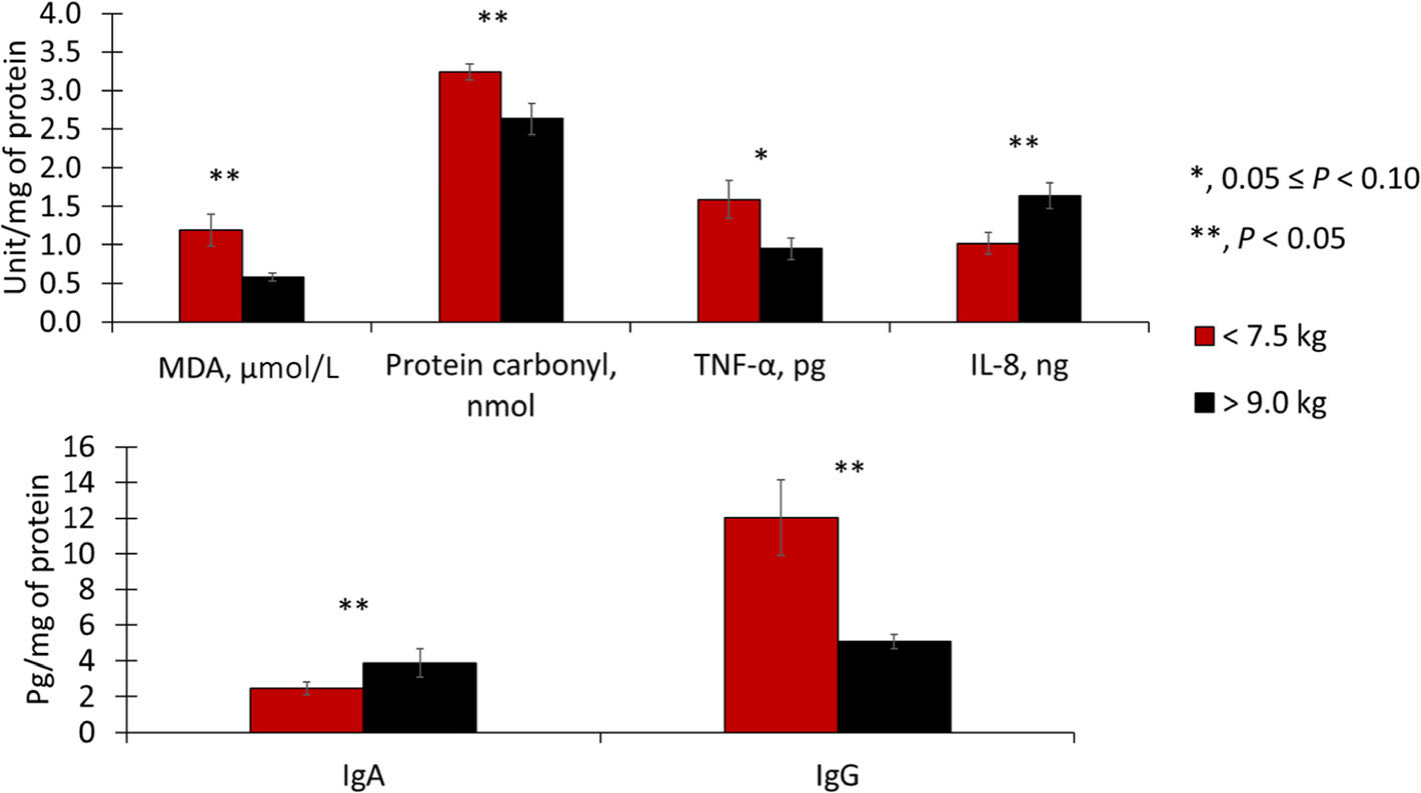
Jejunal oxidative stress, immune markers in pigs with different body weights at weaning. MDA, malondialdehydes; TNF-α, tumor necrosis factor-alpha; IL-8, interleukin 8; IgA, immunoglobulin A; IgG, immunoglobulin G, VH, villus height; CD, crypt depth

On d 0, light pigs tended to have increased (*P* = 0.085) malondialdehydes in duodenal mucosa and had increased (*P* < 0.05) malondialdehydes in jejunal mucosa (Figs. 1 and 2). Light pigs had reduced (*P* < 0.05) interleukin 8 in duodenal mucosa but increased (*P* < 0.05) interleukin 8 in jejunal mucosa. Light pigs had increased (*P* < 0.05) immunoglobulin G in duodenal and jejunal mucosa. Light pigs had increased (*P* < 0.05) protein carbonyls in jejunal mucosa. Light pigs tended to have increased (*P* = 0.055) tumor necrosis factor-α, but tended to have reduced (*P* = 0.090) immunoglobulin A.

**Fig. 3:**
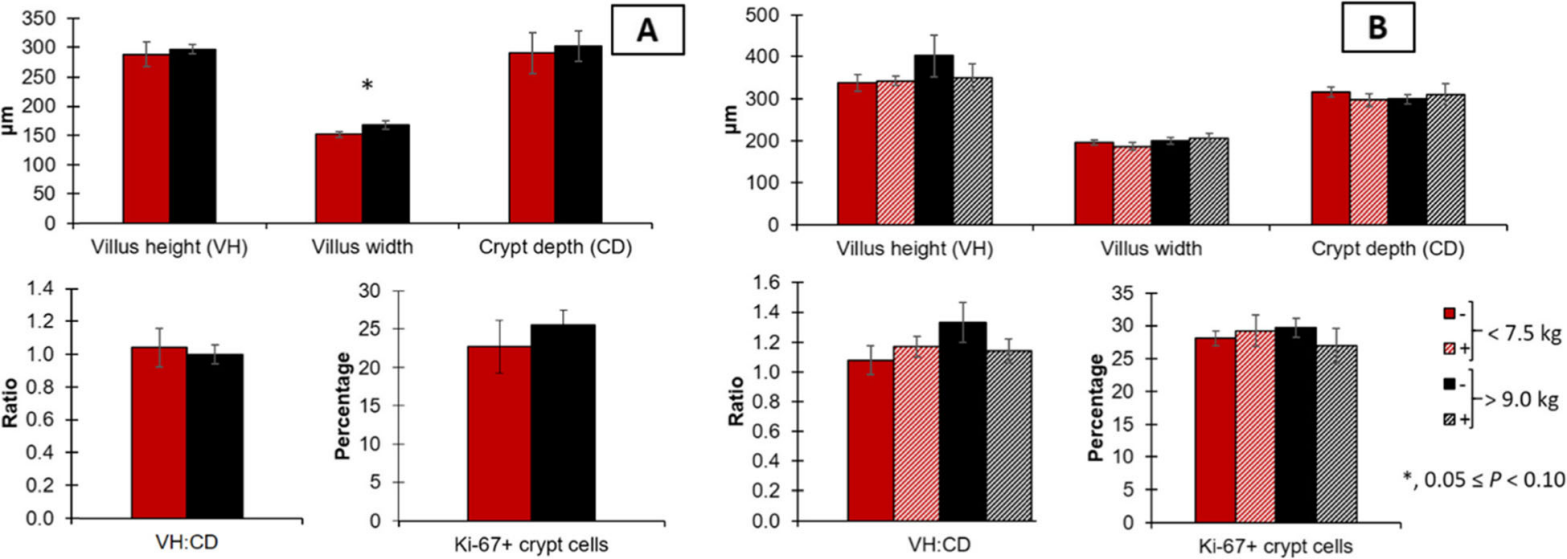
Duodenal morphology in pigs at weaning with different body weights (A) and at 20 kg with different weaning weights (< 7.5 or > 9.0 kg) and mycotoxin challenges (B). (−), no supplemental mycotoxins; (+), supplemental 2 mg/kg of deoxynivalenol and 0.18 mg/kg of aflatoxins; VH, villus height; CD, crypt depth. Ki-67 is an estimate of the proliferative rate, calculated based on the proportion of nuclei positive to Ki-67 staining to the total nucleus number

**Fig. 4:**
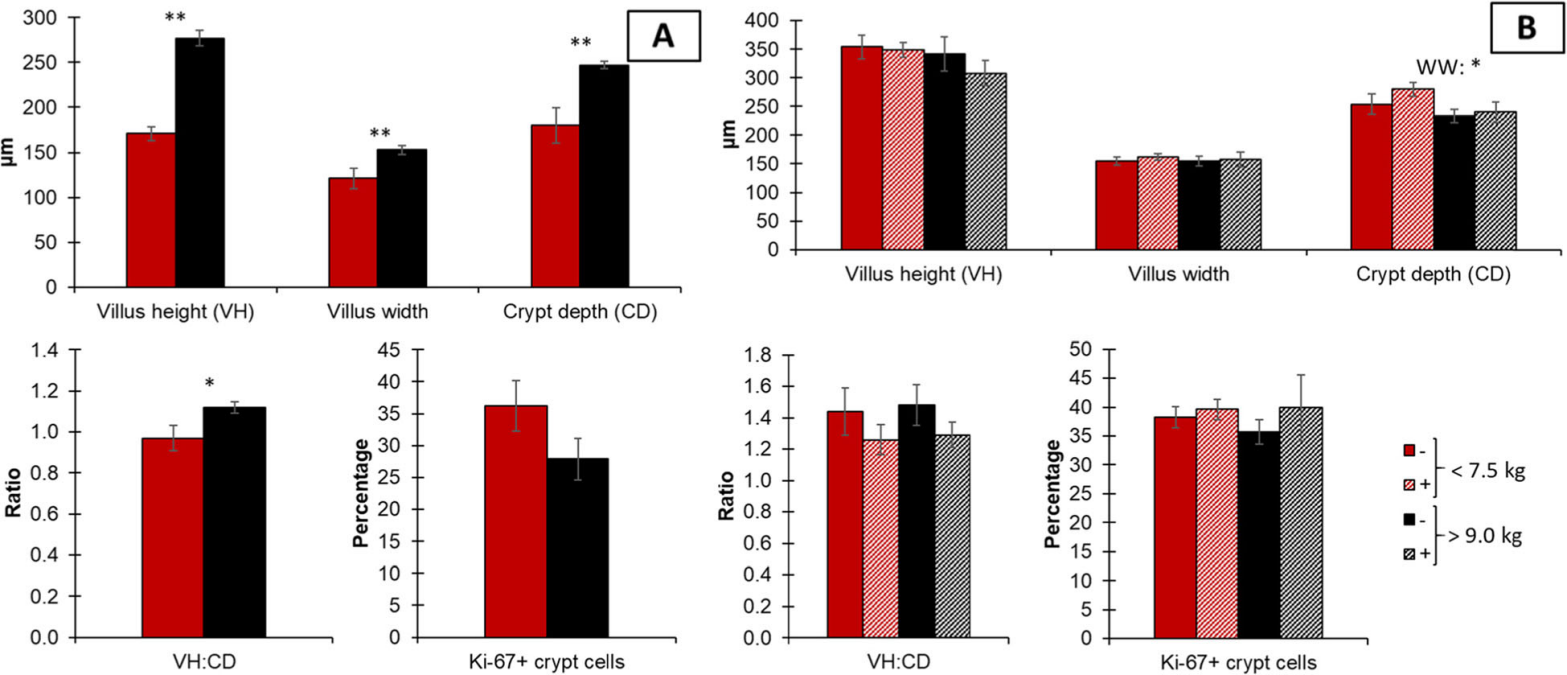
Jejunal morphology in pigs at weaning with different body weights (**A**) and at 20 kg with different (< 7.5 or > 9.0 kg) and mycotoxin challenges (**B**). (−), no supplemental mycotoxins; (+), supplemental 2 mg/kg of deoxynivalenol and 0.18 mg/kg of aflatoxins; VH, villus height; CD, crypt depth. Ki-67 is an estimate of the proliferative rate, calculated based on the proportion of nuclei positive to Ki-67 staining to the total nucleus number. * 0.05 ≤ *P* < 0.1; ** *P* < 0.05

Regarding intestinal morphology on d 0, light pigs tended to have reduced (*P* = 0.089) villus width in the duodenum and had reduced (*P* < 0.05) villus width in the jejunum (Figs. 3 and 4). Light pigs had reduced (*P* < 0.05) villus height, crypt depth, and tended to have reduced (*P* = 0.059) villus height to crypt depth ratio. At 20 kg of body weight, there was no difference (*P* > 0.05) among morphological variables in the duodenum nor the jejunum, except for crypt depth in the jejunum. Light pigs tended to have increased (*P* = 0.053) crypt depth.

**Fig. 5:**
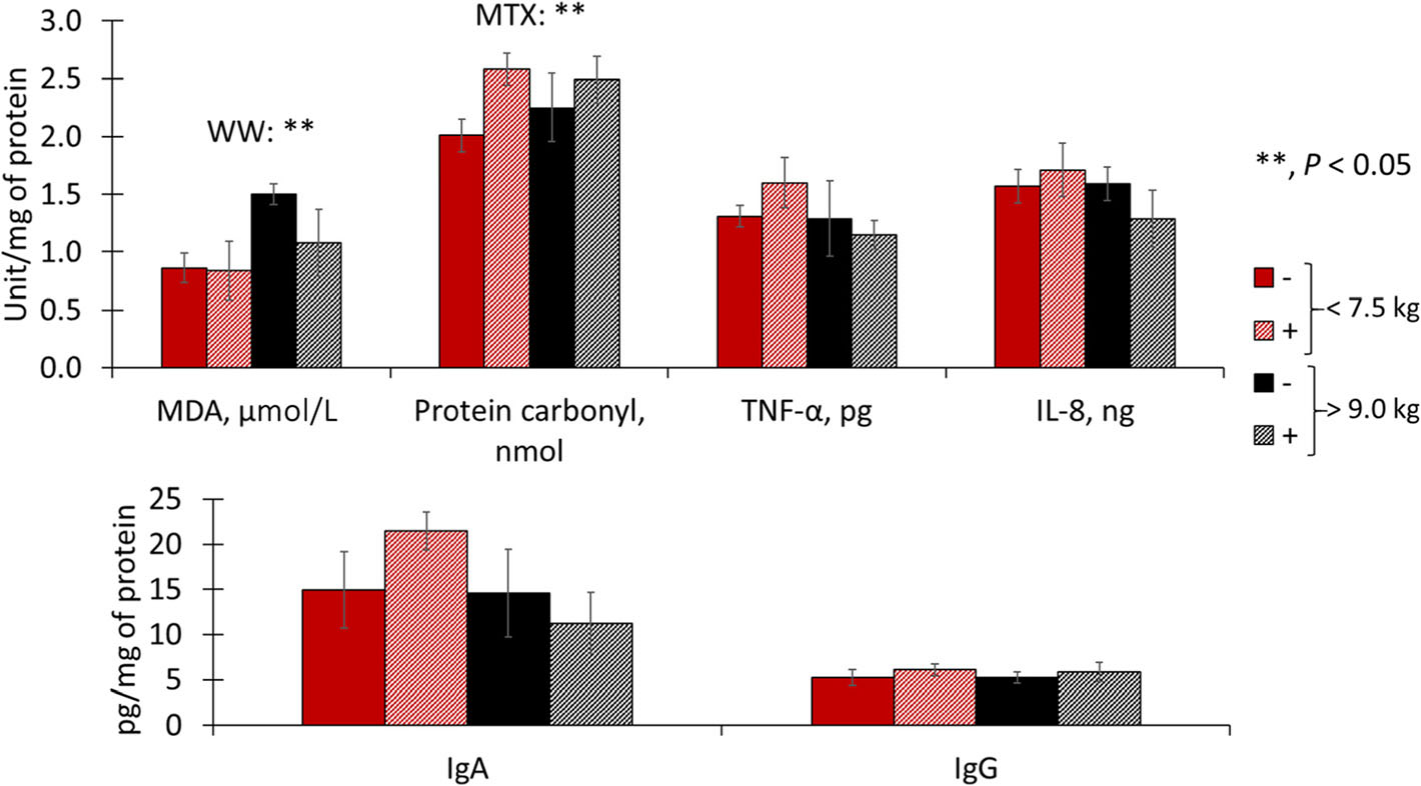
Duodenal Oxidative stress, immune markers in pigs at 20 kg with different weaning weights (< 7.5 or > 9.0 kg) and mycotoxin challenges. (−), no supplemental mycotoxins; (+), supplemental 2 mg/kg of deoxynivalenol and 0.18 mg/kg of aflatoxins; MDA, malondialdehydes; TNF-α, tumor necrosis factor-alpha; IL-8, interleukin 8; IgA, immunoglobulin A; IgG, immunoglobulin G

**Fig. 6:**
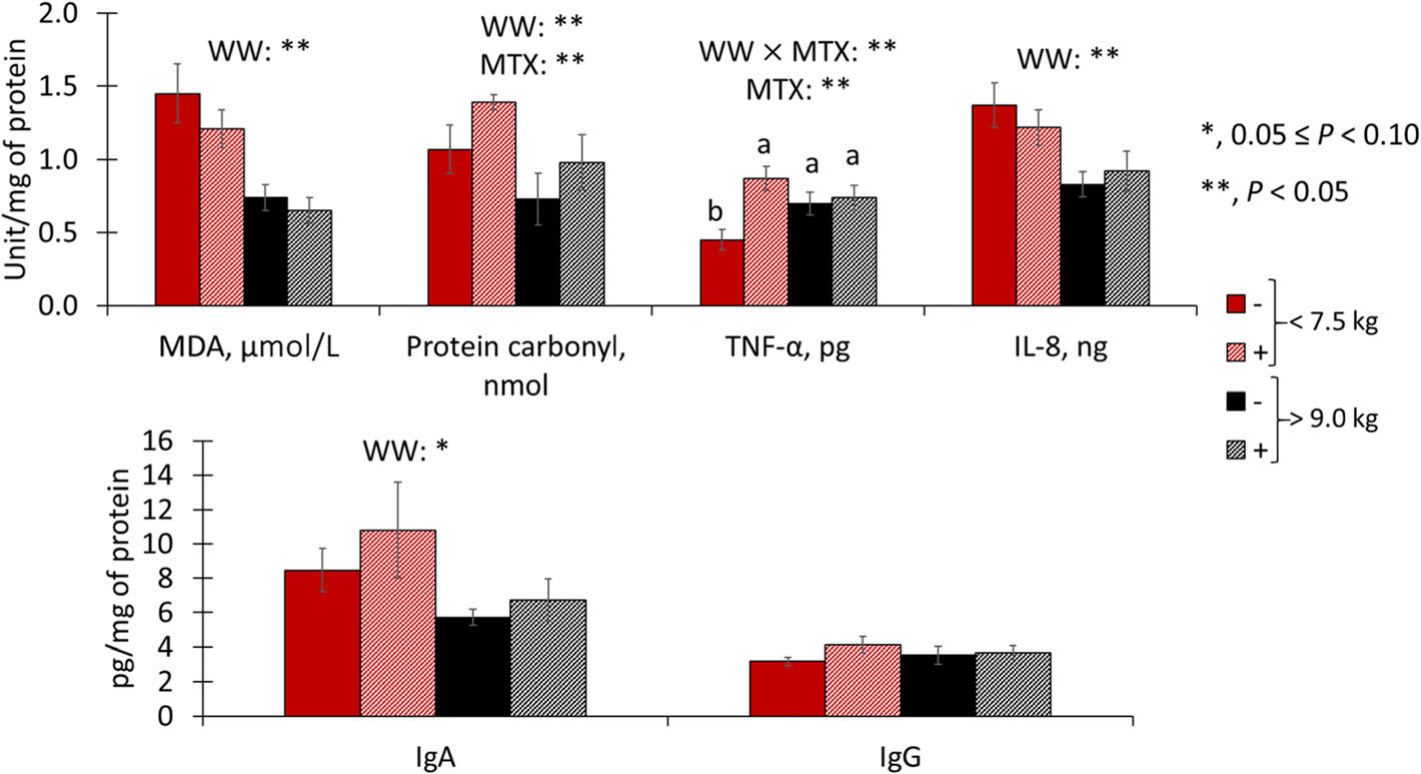
Jejunal Oxidative stress, immune markers in pigs at 20 kg with different weaning weights (< 7.5 or > 9.0 kg) and mycotoxin challenges. (−), no supplemental mycotoxins; (+), supplemental 2 mg/kg of deoxynivalenol and 0.18 mg/kg of aflatoxins; MDA, malondialdehydes; TNF-α, tumor necrosis factor-alpha; IL-8, interleukin 8; IgA, immunoglobulin A; IgG, immunoglobulin G. ^a, b^, means with a different superscript differ (*P* < 0.05)

At 20 kg of body weight, light pigs had reduced (*P* < 0.05) malondialdehydes in duodenal mucosa but increased (*P* < 0.05) malondialdehydes in jejunal mucosa (Figs. 5 and 6). Pigs fed mycotoxins had increased (*P* < 0.05) protein carbonyls in duodenal mucosa and tended to have increased (*P* = 0.080) protein carbonyls in jejunal mucosa. Also, light pigs had increased (*P* < 0.05) protein carbonyls in jejunal mucosa. There was an interaction (*P* < 0.05) for tumor necrosis factor-α in jejunal mucosa, where among light pigs feeding mycotoxins increased (*P* < 0.05) tumor necrosis factor-α concentration, whereas there was no difference among heavy pigs. Light pigs had increased (*P* < 0.05) interleukin 8 and tended to have increased (*P* = 0.055) immunoglobulin A in jejunal mucosa.

**Fig. 7:**
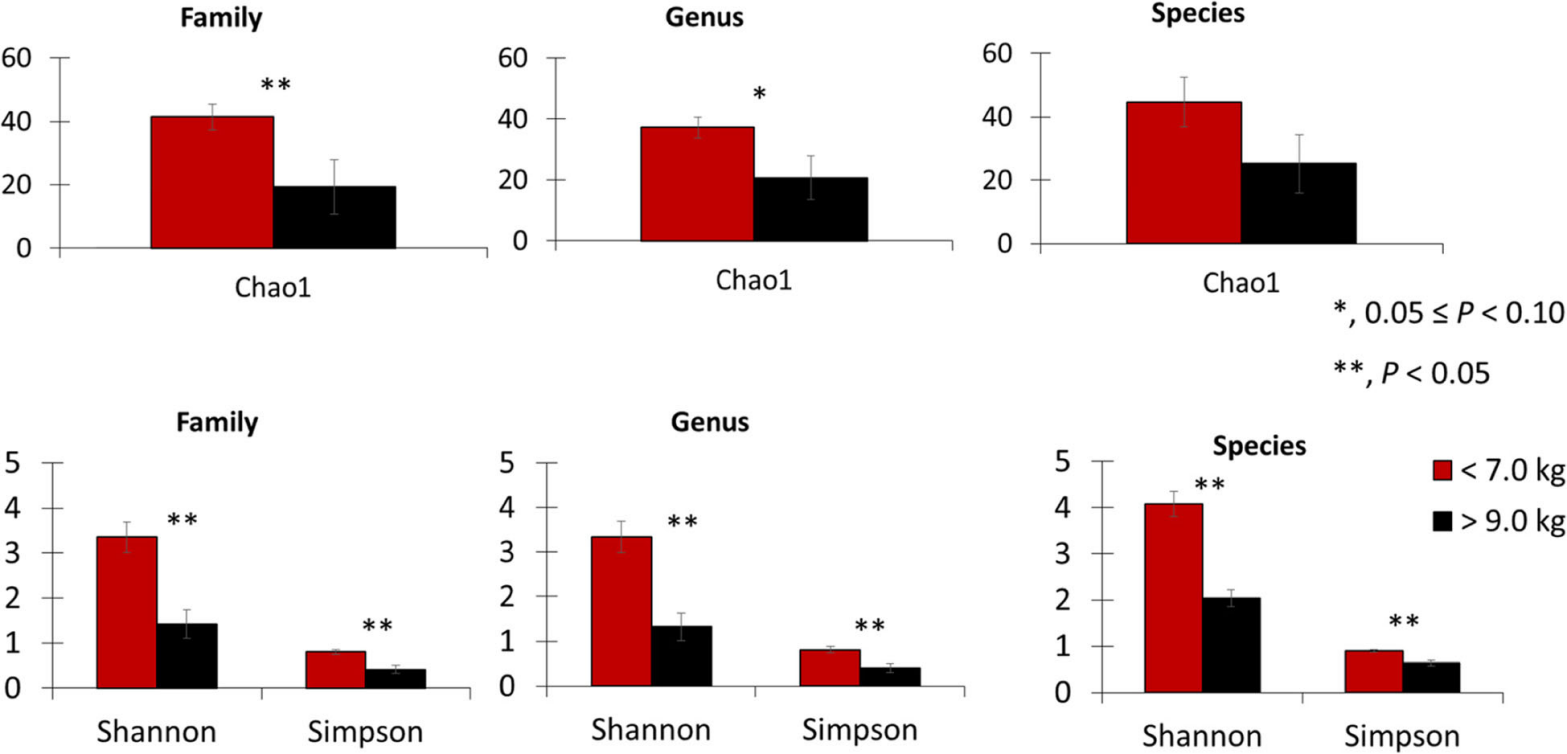
α-diversity indexes of jejunal mucosa-associated microbiota in pigs at weaning with different body weights

**Fig. 8:**
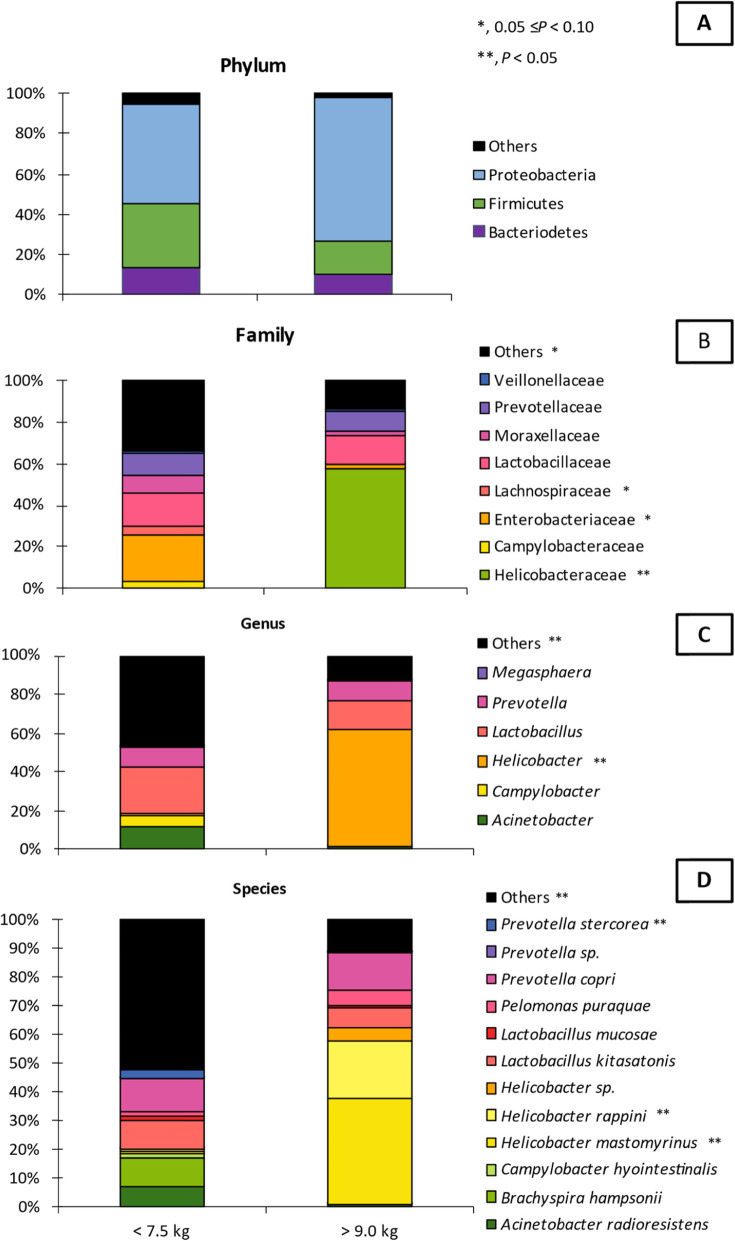
Relative abundance of jejunal mucosa-associated microbiota in pigs at weaning with different body weights for phylum (**A**), family (**B**), genus (**C**), and species (**D**) level. All phyla, families, genera, and species that did not represent 1% of average bacterial diversity among all samples were grouped under the “Others” category

The proximal jejunum microbiota sequencing on d 0 showed that light pigs had increased (*P* < 0.05) α-diversity at the family level according to Chao1, Shannon, and Simpson indexes (Fig. 7). At the genus level, light pigs had increased (*P* < 0.05) α-diversity according to Shannon and Simpson indexes, and tended to have increased (*P* = 0.071) α-diversity for the Chao1 index. At the species level, light pigs had increased (*P* < 0.05) α-diversity according to Shannon and Simpson indexes but no difference was observed for the Chao1 index. The microbiota assessment showed no difference (*P* > 0.05) in relative abundance at phylum level among pigs at weaning (Fig. 8). Light pigs had decreased (*P* < 0.05) relative abundance of Helicobacteriaceae. On the other hand, light pigs tended to have an increased relative abundance of Enterobacteriaceae (*P* = 0.063), Lachnospiraceae (*P* = 0.067), and the sum of families whose individual relative abundances were less than 1% (*P* = 0.094), reported as “Others” in the current study. There was no difference in the families of Campylobacteraceae, Lactobacillaceae, Moraxellaceae, Prevotellaceae, and Veillonellaceae among pigs. Light pigs had reduced (*P* < 0.05) relative abundance for *Helicobacter* but increased (*P* < 0.05) relative abundance for the sum of genera in which individual relative abundances were less than 1%, reported as “Others” in the current study. There was no difference in the relative abundance for *Acinetobacter*, *Campylobacter*, *Lactobacillus*, *Megasphaera*, and *Prevotella* among pigs. Light pigs had reduced (*P* < 0.05) relative abundance for *Helicobacter mastomyrinus* and *Helicobacter rappini* but increased (*P* < 0.05) for *Prevotella stercorea*. Light pigs had increased (*P* < 0.05) relative abundance for the sum of species whose individual relative abundances were less than 1%, reported as “Others” in the current study. There was no difference (*P* > 0.05) in the relative abundance for *Acinetobacter radioresistens*, *Brachyspira hampsonii*, *Campylobacter hyointestinalis*, *Helicobacter* sp., *Lactobacillus kitasatonis*, *Lactobacillus mucosae*, *Pelomonas puraquae*, *Prevotella copri*, and *Prevotella* sp. among pigs.

**Fig. 9:**
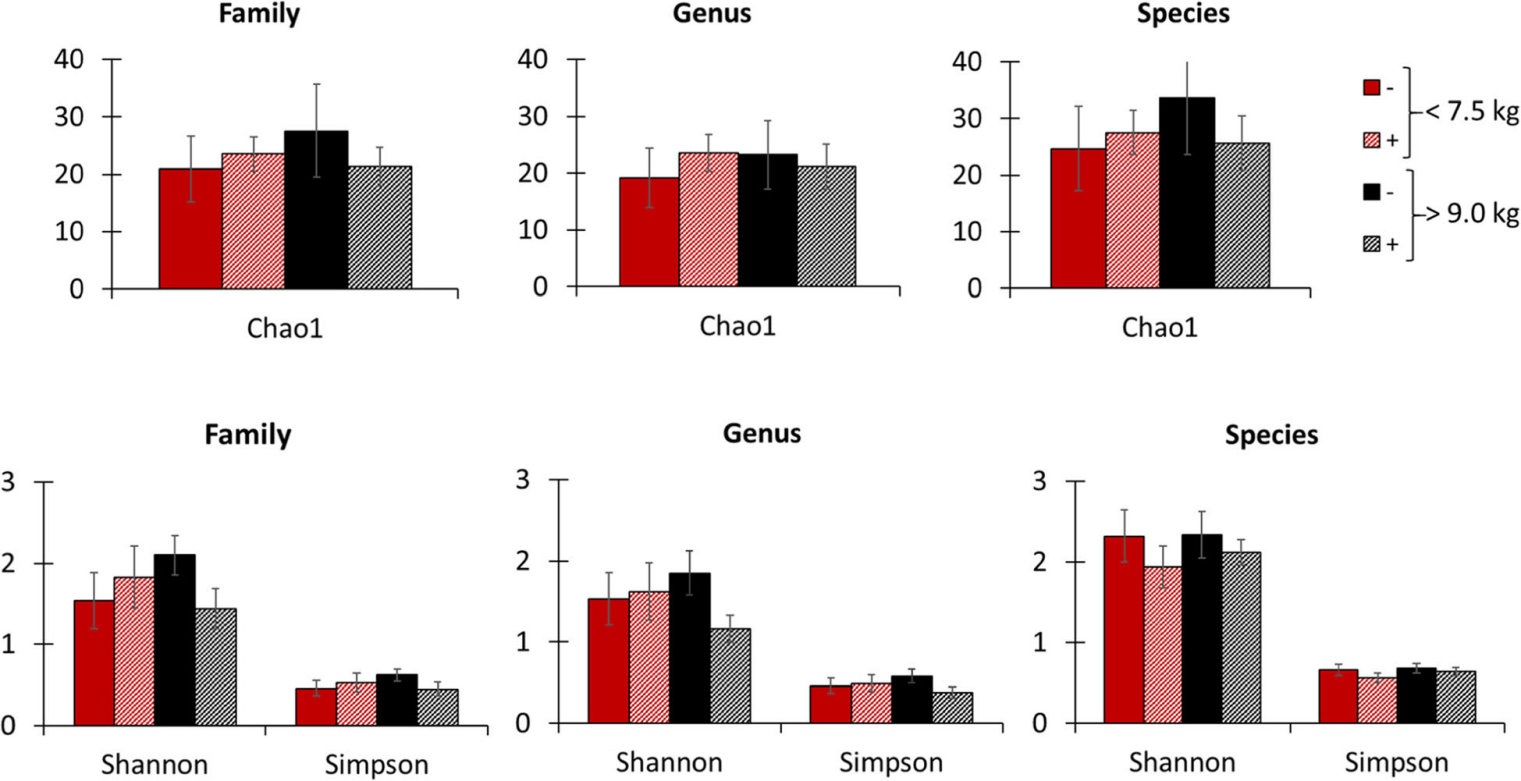
α-diversity indexes of jejunal mucosa-associated microbiota in pigs at 20 kg with different weaning weights (< 7.5 or > 9.0 kg) and mycotoxin challenges. (−), no supplemental mycotoxins; (+), supplemental 2 mg/kg of deoxynivalenol and 0.18 mg/kg of aflatoxins

At 20 kg of body weight, there was no difference (*P* > 0.05) in the α-diversity indexes assessed for the family level among pigs (Fig. 9). Also, no differences (*P* > 0.05) were observed in the α-diversity indexes assessed for either genus nor species levels among pigs.

**Fig. 10:**
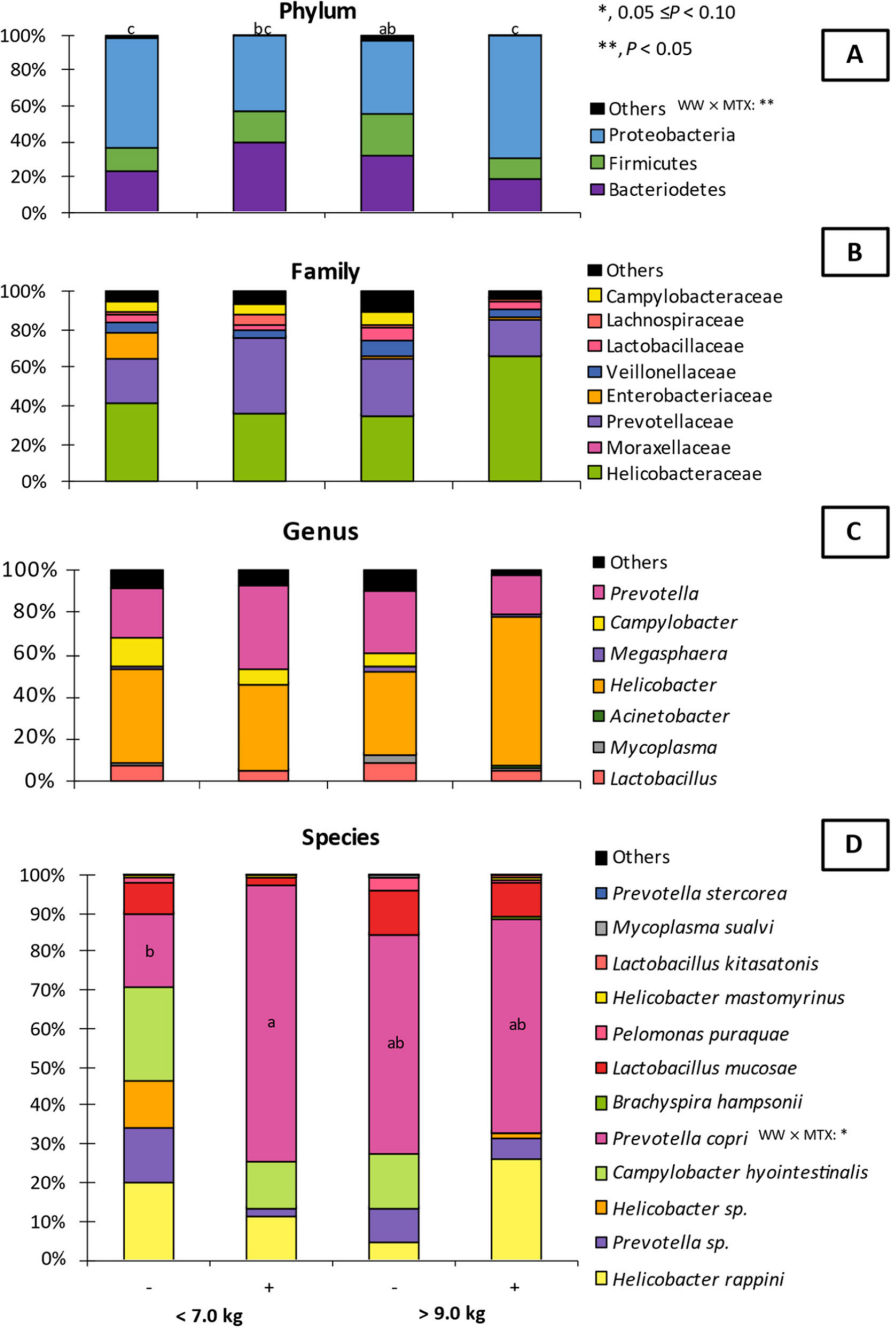
Relative abundance of jejunal mucosa-associated microbiota in pigs at 20 kg with different weaning weights (< 7.5 or > 9.0 kg) and mycotoxin challenges for phylum (**A**), family (**B**), genus (**C**), and species (**D**) level. All phyla, families, genera, and species that did not represent 1% of average bacterial diversity among all samples were grouped under the “Others” category. ^a, b, c^, means with a different superscript differ (*P* < 0.05). (−), no supplemental mycotoxins; (+), supplemental 2 mg/kg of deoxynivalenol and 0.18 mg/kg of aflatoxins

The microbiota assessment showed no differences (*P* > 0.05) in bacteria abundance at phylum level among pigs at 20 kg, except for Proteobacteria, which showed a tendency (*P* = 0.071) for interaction (Fig. 10). However, no differences (*P* > 0.10) were observed regarding Proteobacteria comparisons among pig. It was observed an interaction (*P* < 0.05) for the relative abundance of Others, where among heavy pigs feeding mycotoxins reduced (*P* < 0.05) the abundance of Others, whereas there was no difference among light pigs. There was no difference in the relative abundance for Helicobacteraceae, Moraxellaceae, Prevotellaceae, Enterobacteriaceae, Veillonellaceae, Lactobacillaceae, Lachnospiraceae, and Campylobacteraceae among pigs. There was no difference in the relative abundance for *Lactobacillus*, *Mycoplasma*, *Acinetobacter*, *Helicobacter*, *Megasphaera*, *Campylobacter*, *Prevotella*, and Others among pigs. There was a tendency for interaction (*P* = 0.066) where among light pigs feeding mycotoxins increased (*P* < 0.05) the relative abundance for *Prevotella copri*, whereas there was no difference among heavy pigs. There was no difference (*P* > 0.05) in the relative abundance for *Helicobacter rappini*, *Prevotella* sp., *Helicobacter* sp., *Campylobacter hyointestinalis*, *Brachyspira hampsonii*, *Lactobacillus mucosae*, *Pelomonas puraquae*, *Helicobacter mastomyrinus*, *Lactobacillus kitasatonis*, *Mycoplasma sualvi*, *Prevotella stercorea*, and Others among pigs.

## Discussion

There was a difference observed in weaning weight on d 0 within the animals used in this study. Previous researchers demonstrated that such difference can be due to different birth weight, weaning ages [6], or the use of artificial nursing methods during lactation [29]. However, differences in birth weight were probably masked by the considerable number of pigs used in the current study (*n* = 96). Also, all the pigs used in the current study were not provided any supplemental nutritional sources during the nursing period besides to sow milk. Therefore, such a difference in weaning weight was likely caused by differences in weaning age. Nonetheless, weaning ages were not recorded in this study, thus, the weaning weight difference will be the term used in the current study.

Because the pigs used in the current study had different initial body weights, growth performance data are shown by phase, to allow the comparison among factors and treatments. The difference in the initial body weight made light pigs spend 10 additional days to achieve the targeted body weight, 20 kg, by the end of the study. Phase 1 diet was fed from d 0 until pigs achieved 11 kg (4 d for heavy pigs and 14 d for light pigs), whereas for phase 2 both weaning weight groups took the same time, 17 d. The difference in body weight among weaning weight groups was not expected by the end of phase 1. The targeted final body weight was set based on the time expected to elicit mycotoxin effects in the most recent and studies from our research team and nursery pigs with similar weaning weights, approximately 15 to 21 d [11, 14].

The negative effect of feeding 2 mg/kg of deoxynivalenol and 0.2 mg/kg of aflatoxins on growth performance was observed by the end of the study with an average reduction of 2.1 kg in pig body weight. Light pigs had reduced ADFI during phase 1, which could be explained by difficulties in adaptation to a solid and plant-based diet after weaning leading to reduced ADG during phase 1. The effects of weaning age on ADFI and ADG of pigs are not new. It has been demonstrated that ADFI and ADG of nursery pigs are directly proportional to weaning age [3]. However, during phase 2, light pigs seemed to show a recovery in both ADFI and ADG. Such result could be related to the 10-day-longer phase 1 period for light pigs in comparison to heavy pigs (14 vs. 4 d, respectively), thus, light pigs had more time to develop their gastrointestinal tract and were not challenged to the same extent by phase 2 diet formulation (with increased soybean meal percentage and reduced milk-derived and animal-based ingredients). It is important to note that the reduction in overall ADFI (17%) caused by mycotoxins is likely the sole cause for the reduction observed in ADG (15%). The current study did not observe further toxic effects of mycotoxins reducing nutrient utilization by pigs as also observed by Holanda et al. [15] when challenging nursery pigs with dietary deoxynivalenol (2.0 mg/kg of diet) and aflatoxin (0.2 mg/kg of diet). Instead, it was observed that pigs fed mycotoxins had reduced ADFI and ADG, but increased G/F, which could be an adaptation to better utilize the comparatively reduced amount of energy and nutrients ingested [30]. Nevertheless, a comparable study conducted by Weaver et al. [31] fed weaned pigs either 0 or 6% spray-dried plasma protein resulting in pigs with about 1 kg difference at the beginning of phase 2 (9.0 vs. 9.9 kg, respectively). During phase 2, the light group of pigs showed a reduction in body weight and ADFI upon mycotoxin challenge, whereas no differences were observed among the heavy group of pigs. However, the dietary treatment received during phase one (0 or 6% spray-dried plasma protein) may have influenced the outcomes observed relative to mycotoxin susceptibility.

The increased fecal score observed on d 0, 10, and 20 in light pigs could be due to their lower maturity of gastrointestinal tract. As demonstrated in a previous study [4], pigs with lighter body weight showed an increased duration of diarrhea caused by weaning stress. Regarding the interaction observed for the fecal score on d 15, light pigs presented increased fecal scores in comparison to heavy pigs following the same reason discussed before. There was a mycotoxin effect, however, to increase the fecal score of light pigs fed mycotoxins in comparison to heavy pigs which were not fed mycotoxins, whereas there were no differences to heavy pigs fed mycotoxins. An increased fecal score or diarrhea incidence is related to impaired intestinal health [32]. Thus, mycotoxins may have had impaired the health of the gastrointestinal tract of pigs and light pigs may be more susceptible to such mycotoxin toxic effects [31]. Supporting the toxic effects of mycotoxins in the gastrointestinal tract, mycotoxins caused a decrease in apparent ileal digestibility of dry matter and protein in the current study. It was previously reported a decrease in apparent ileal digestibility of dry matter and protein in nursery pigs fed aflatoxins and deoxynivalenol [15]. Growing pigs also showed reduced apparent total tract digestibility of dry matter and nitrogen in feed under the mycotoxin challenge [16]. The reduced gastrointestinal health and digestibility caused by mycotoxins could be caused by the impairment of cell metabolism. Deoxynivalenol has an inhibitory effect over protein synthesis at the cytoplasm through a mechanism known as ribotoxic stress [33]. As reviewed by Pestka [34], the ribotoxic stress happens when deoxynivalenol interacts with ribosomes hindering protein translation at the same time as leading to downstream activation of mitogen-activated protein kinases (MAPK) and ultimately inducing immune response and cell apoptosis. By such mechanism of impaired protein synthesis, deoxynivalenol may hinder sodium-dependent glucose transporter 1 expression and function [35, 36]. Moreover, an increase in cell apoptosis can reduce the efficiency of nutrient uptake and shift the utilization of nutrients for tissue repair or towards the immune response stimulated by MAPK activation. On the other hand, aflatoxins inhibit messenger RNA synthesis impairing the activity of RNA polymerase at the nucleus [37]. However, feeding mycotoxins increased lipid digestibility among light pigs in the current study. It was previously shown that lipid digestibility increases as pigs grow after weaning [3]. Thus, the increased lipid digestibility could be due to the extended duration after weaning as light pigs achieved 20 kg of body weight in 31 d after weaning, whereas heavy pigs took 21 d. Another factor could be the reduced body weight in pigs fed mycotoxins, as smaller animals are more efficient in utilizing nutrients from feeds [30].

Even though no blood was collected at the beginning of the study and differences observed at 20 kg cannot be compared to pre-existing differences due to weaning weight, the increased alkaline phosphatase in light pigs may suggest increased enterocyte differentiation to support growth. Alkaline phosphatase is indicative of enterocyte differentiation and functionality and its increase is linked to increased digestive and absorptive function [38, 39]. Indeed, light pigs presented increased ADFI and ADG during phase 2. Light pigs had reduced chloride levels than heavy pigs. It is possible that light pigs were not absorbing chloride as effectively as heavy pigs or that there was an electrolyte leakage into the intestinal lumen. However, it is important to highlight that the difference observed was of 3 mEq/L and further investigations would be necessary to elucidate chloride absorption between light and heavy weaned pigs. The poor absorption or electrolyte leakage could be caused by increased oxidative stress and local immune activation, which may have increased intestinal permeability [40] as seen by the increased fecal score. However, no differences were observed for other electrolytes. Mycotoxins, as also seen in other variables measured, likely impaired protein synthesis decreasing albumin concentration and albumin to globulin ratio. Albumin is the main blood serum protein and, thus, can be used as an estimate of protein synthesis and hepatic function [41]. Interactions of weaning weight and mycotoxin factors showed that there was an increase in globulin in light pigs. Such an outcome is contradictory to the initial hypothesis and the outcomes observed for other variables (albumin, oxidative stress markers, nutrient digestibility, morphological measurements), showing the impairment over protein function caused by mycotoxins [8, 9]. Creatinine was increased among heavy pigs fed mycotoxins, decreasing the blood urea nitrogen to creatinine ratio in these animals. Creatinine is indicative of hepatic function, where its increase could be related to hepatic damage and failure [17] caused by mycotoxins. Concerning the effects on electrolytes, the negative effect of aflatoxins on bone ossification because of its impact on vitamin D and calcium metabolism was previously shown [42]. The strict equilibrium maintained among calcium and phosphorus could have caused the reduction observed phosphorus concentration in heavy pigs fed mycotoxins. Nevertheless, no differences were observed among calcium serum levels.

On d 0, immune response and oxidative stress markers were greatly affected by weaning weight in the jejunum rather than in the duodenum. In the duodenum, light pigs had increased malondialdehydes, indicating lipid peroxidation caused by oxidative stress [10]. Among immune response markers, light pigs had reduced interleukin 8 but increased immunoglobulin G, suggesting reduced cellular but increased humoral immune response [43, 44]. The villus width in light pigs was also smaller in comparison to heavy pigs at weaning. Supporting the outcomes observed in the duodenum, the results from the jejunum showed a similar trend but with more pronounced effects. Both increased concentrations of malondialdehydes and protein carbonyls in light pigs are indicative of oxidative stress exceeding the antioxidative capacity of enterocytes [10]. The increased oxidative stress was likely caused by the increased inflammatory response, represented by the increased tumor necrosis factor-α [1]. In the jejunum, a similar pattern was found where light pigs had decreased interleukin 8 and increased immunoglobulin G in the duodenum, reinforcing a diminished local immune activation and, instead, a more pronounced systemic immune response [43]. Also, light pigs had a reduced concentration of immunoglobulin A stressing the reduced recruitment of local immune cells [43]. Taking together duodenum and jejunum measurements, light pigs seemed to be more susceptible to stress factors of weaning, showing increased inflammation, oxidative stress, and presenting increased local cellular immune response. The increased inflammation, oxidative stress, and systemic local immune response resulted in enterocyte damage and death. The loss of enterocytes can be inferred by the equivalent number of proliferating cells in crypts as measured by the percentage of Ki-67^+^ cells between the body weight groups. This suggests that light pigs were not able to replace enterocytes damaged by weaning stress and resulted in decreased intestinal surface [1] (villus width, villus height, crypt depth, and villus height to crypt depth ratio) in light pigs. Light pigs, in comparison to heavy pigs, showed increased intestinal oxidative stress and inflammation, and systemic immune response activation on d 0 along with increased fecal score during the experimental period. Such outcomes indicate that light pigs experienced more severe weaning stress than heavy pigs in the current study.

At 20 kg of body weight, like the outcomes observed on d 0, the immune response, oxidative stress markers, and morphology were more influenced by weaning weight and mycotoxin factors in the jejunum than in the duodenum. In the duodenum, a reduced concentration of malondialdehydes showed in light pigs was unexpected, especially when put aside to the increased oxidative stress markers (malondialdehydes and protein carbonyls) in the jejunum of light pigs. Mycotoxins increased protein carbonyls in the duodenum suggesting increased oxidative stress [10]. The fact that only proteins were affected by oxidative stress could be due to the impaired RNA transcription and translation caused by aflatoxins and deoxynivalenol [33, 37]. Therefore, lipids may have had the ability to be repaired or replaced, as seen by no differences observed in malondialdehydes in the current study, whereas it did not happen to the same extent for proteins. In the jejunum, the increased malondialdehydes and protein carbonyls in light pigs suggest that light pigs were experiencing a greater and long-lasting challenge 31 d after weaning than heavy pigs 21 d after weaning. Different from what was observed on d 0, light pigs seemed to have more pronounced local immune activation, as seen by the greater concentrations of interleukin 8 and immunoglobulin A [43, 44]. The detrimental effects of weaning stress and mycotoxin challenge in light pigs caused an increment in crypt depth. Suggesting an increased cell proliferation to provide enterocytes to maintain villus height [1] and their digestive, absorptive, and barrier function. Also, the lack of difference in Ki-67^+^ cell counting along with the difference in crypt depth observed in the current study highlights the need to combine the outcome of both measurements when assessing enterocyte proliferation, as the first is a proportion of actively proliferating cells in a given moment and the latter is an estimate of the number of cells with the potential to multiply. Mycotoxins increased oxidative stress in the jejunum, based on the increase in protein carbonyls [10]. Of note, light pigs may be more prone to inflammatory effects caused by mycotoxins as light pigs fed mycotoxins showed increased tumor necrosis factor-α, whereas no differences were observed among heavy pigs.

At weaning, d 0, α-diversity observed in heavy pigs was smaller than lighter pigs. The reduced α-diversity could be noticed at the family, genus, and species levels, where heavy pigs showed a reduced relative abundance of ‘Others’ under family, genus, and species levels, indicating that a smaller proportion of the microbiota is composed of diversified bacteria. After weaning with a start of feed consumption, α-diversity of microbiota in pigs decreases [7], followed by a steady and slow increase until pigs reached market weight. It is speculated that heavy pigs at weaning may had greater access to sow feeds during suckling period which caused reduced α-diversity compared with lighter pigs at weaning. Weaned pigs were fed diets containing zinc in the referred study [7], whereas there was no inclusion of zinc in the nursery diets in the current study because of its effect reducing microbiota diversity in the post-weaning period [45]. Also, an increased relative abundance was observed for *Prevotella stercorea* in light pigs. Such a result is in agreement with the increased relative abundance from 11 to 20 days of age for *P. stercorea* in nursing piglets [7]. The decreased relative abundance of Helicobacteriaceae for light pigs was followed by decreased relative abundances for *Helicobacter* as well as for *H. mastomyrinus* and *H*. *rappini*. This outcome was unexpected, as bacteria from the *Helicobacter* are commonly recognized as opportunistic pathogens [46]. Also, light pigs showed increased proportions of their microbiota from Enterobacteriaceae and Lachnospiraceae. Similar outcomes were reported by Frese et al. [47], where Enterobacteriaceae and Lachnospiraceae families were the ones with increased abundance in nursing piglets. However, the same study demonstrated that Enterobacteriaceae had its relative abundance decreased after weaning and as animals matured, whereas Lachnospiraceae remained relatively constant. In nursery pigs, an increased relative abundance in Lachnospiraceae was related to increased ADFI [48]. Even though milk consumption by pigs was not estimated in the current study, it can be inferred that light pigs were likely to be consuming a reduced amount of milk in comparison to heavy pigs. Thus, in the current study, the positive relationship between feed intake and Lachnospiraceae abundance was not sustained in the case of nursing piglets.

The influence of feed change, from sow milk to solid feed, on intestinal microbiota was reported to start as early as 8 d after weaning [7]. Hence, the dietary treatments provided for 31 or 21 d for light or heavy pigs, respectively, were expected to influence the relative abundance of microbiota assessed in the current study. The increased relative abundance for *P. stercorea* observed in light pigs on d 0 disappeared at 20 kg of body weight, a similar reduction was observed in a longitudinal study when comparing pigs sampled before weaning and during the nursery period [7]. Regarding the increase in the relative abundance for *P. copri* during the nursery phase in light pigs fed mycotoxins, a similar outcome was described by Wang et al. [7] with an increase in *Prevotella* in the cecum and colon of weaned pigs fed deoxynivalenol for 28 d (versus 31 d for light pigs in the current study). Also, an increased abundance of *P. copri* was observed right after the introduction of solid feed [7]. However, there seems to be a decrease for *Prevotella* with age [49], suggesting that the increase can be related to the change to a solid and plant-based diet and the following decrease could be related to the establishment of the gut microbial community. Comparing nursing piglets and nursery pigs, Frese et al. [47] reported an increased relative abundance of *Prevotelaceae* in nursery pigs. Suggesting that changes in *Prevotelaceae* abundance can be related to the diet [7], instead of the animal age during the nursing period as piglets showed no difference until 28 days of age [47]. In the current study, there were no differences in the family or genus levels at 20 kg. The increased relative abundance for *P. copri* among light pigs fed mycotoxins at 20 kg of body weight may indicate that light pigs fed mycotoxins took an additional time to establish their microbial community in comparison to heavy pigs and light pigs fed the diet without additional mycotoxins. Similar to the findings in the current study, *P. stercorea* has been found to have increased abundance in nursing pigs, whereas *P. copri* has been found to have increased abundance during the nursery phase even though both species belong to *Prevotella* [7]. However, the lack of difference in the α-diversity index at 20 kg of body weight may indicate that both light and heavy pigs have achieved similar maturation levels in their respective microbial communities in the intestine and, therefore, they did not differ at this time point. Likewise, challenging nursery pigs with deoxynivalenol (8 mg/kg of feed) did not result in differences in microbial diversity in the gut using the same index tested in the current study (Chao1 and Shannon) and the phylogenetic diversity whole tree [50]. Despite the previously described differences in microbiota diversity indexes and relative abundance, the current study did not observe any difference among pigs for phylum level at weaning or at 20 kg. Such result can be explained by the limited categories of phylum that compose the majority of gut microbiota. It has been reported that Firmicutes and Bacteriodetes commonly account for > 50% of relative abundance among all bacterial phyla in pigs [51–53].

Taking the increased proportion of *P. copri* in light pigs fed mycotoxins and the absence of difference in the α-diversity indexes may suggest that *P. copri* can serve as a single, and perhaps, more precise indicator than the overall microbiota diversity regarding microbiota community establishment particularly in the case of mycotoxin challenge. The pig microbiota was expected to stabilize, reducing the variability as pigs aged [49].

## Conclusion

Dietary mycotoxin challenge (supplemental aflatoxins at 0.2 mg/kg and deoxynivalenol 2.0 mg/kg of feed) impaired nutrient digestibility, health, and growth performance independent of pig weaning weight. Light pigs (weaning weight <7.5 kg) showed impaired health and immature microbiota in comparison to heavy pigs (weaning weight > 9.0 kg) upon weaning but the microbiota composition became similar to heavy pigs when both achieved 20 kg. Of interest, the proximal jejunum of weaned pigs showed an increased susceptibility to weaning stress and mycotoxin challenge than the duodenum. Current results indicate that the detrimental effects of mycotoxins affect nursery pigs regardless weaning stress magnitude.

## Data Availability

The datasets used and/or analyzed during the current study are available from the corresponding author on reasonable request.

## Abbreviations

ADFI: Average daily feed intake
ADG: Average daily gain
BW: Body weight
G/F: Gain to feed ratio
MTX: Mycotoxins
MAPK: Mitogen-activated protein kinases
OTU: Operational taxonomic unit
SAS: Statistical analysis system
TNF-α: Tumor necrosis factor-α
WW: Weaning weight
